# Abnormal Bone Tissue Organization and Osteocyte Lacunocanalicular Network in Early‐Onset Osteoporosis Due to *SGMS2* Mutations

**DOI:** 10.1002/jbm4.10537

**Published:** 2021-08-20

**Authors:** Riikka E. Mäkitie, Stéphane Blouin, Ville‐Valtteri Välimäki, Sandra Pihlström, Kirsi Määttä, Minna Pekkinen, Nadja Fratzl‐Zelman, Outi Mäkitie, Markus A. Hartmann

**Affiliations:** ^1^ Folkhälsan Institute of Genetics Helsinki Finland; ^2^ Research Program for Clinical and Molecular Metabolism, Faculty of Medicine University of Helsinki Helsinki Finland; ^3^ Department of Otorhinolaryngology–Head and Neck Surgery Helsinki University Hospital and University of Helsinki Helsinki Finland; ^4^ Ludwig Boltzmann Institute of Osteology at Hanusch Hospital of OEGK and AUVA Trauma Centre Meidling, 1st Medical Department Hanusch Hospital Vienna Austria; ^5^ Vienna Bone and Growth Center Vienna Austria; ^6^ Pohjola Hospital Helsinki Finland; ^7^ Children's Hospital, University of Helsinki and Helsinki University Hospital Helsinki Finland; ^8^ Department of Molecular Medicine and Surgery and Center for Molecular Medicine Karolinska Institutet Stockholm Sweden

**Keywords:** BONE HISTOMORPHOMETRY, CONFOCAL LASER SCANNING MICROSCOPY, EARLY‐ONSET OSTEOPOROSIS, QUANTITATIVE BACKSCATTERED ELECTRON IMAGING, SPHINGOMYELIN METABOLISM, SPHINGOMYELIN SYNTHASE 2

## Abstract

Pathological variants in *SGMS2*, encoding sphingomyelin synthase 2 (SMS2), result in a rare autosomal dominant skeletal disorder with cranial doughnut lesions. The disease manifests as early‐onset osteoporosis or a more severe skeletal dysplasia with low bone mineral density, frequent fractures, long‐bone deformities, and multiple sclerotic cranial lesions. The exact underlying molecular features and skeletal consequences, however, remain elusive. This study investigated bone tissue characteristics in two adult males with a heterozygous *SGMS2* mutation p.Arg50* and significant bone fragility. Transiliac bone biopsy samples from both (patient 1: 61 years; patient 2: 29 years) were analyzed by bone histomorphometry, confocal laser scanning microscopy, and quantitative backscattered electron imaging (qBEI). Bone histomorphometry portrayed largely normal values for structural and turnover parameters, but in both patient 1 and patient 2, respectively, osteoid thickness (−1.80 SD, −1.37 SD) and mineralizing surface (−1.03 SD, −2.73 SD) were reduced and osteoid surface increased (+9.03 SD, +0.98 SD), leading to elevated mineralization lag time (+8.16 SD, +4.10 SD). qBEI showed low and heterogeneous matrix mineralization (CaPeak −2.41 SD, −3.72 SD; CaWidth +7.47 SD, +4.41 SD) with a chaotic arrangement of collagenous fibrils under polarized light. Last, osteocyte lacunae appeared abnormally large and round in shape and the canalicular network severely disturbed with short‐spanned canaliculi lacking any orderliness or continuity. Taken together, these data underline a central role for functional SMS2 in bone matrix organization and mineralization, lacunocanalicular network, and in maintaining skeletal strength and integrity. These data bring new knowledge on changes in bone histology resulting from abnormal sphingomyelin metabolism and aid en route to better understanding of sphingolipid‐related skeletal disorders. © 2021 The Authors. *JBMR Plus* published by Wiley Periodicals LLC on behalf of American Society for Bone and Mineral Research.

## Introduction

In recent years, several new forms of monogenic osteoporosis have been identified, each contributing to our understanding of the genetic factors and molecular pathomechanisms governing skeletal health.^(^ ^1^ ^)^ In 2019, we identified mutations in sphingomyelin synthase 2 (*SGMS2*), encoding SMS2, to cause a previously clinically described disorder—osteoporosis with calvarial doughnut lesions (OP‐CDL; OMIM #126550).^(^ ^2^ ^)^ Depending on the underlying *SGMS2* variant, affected subjects present with childhood‐onset osteoporosis with low bone mineral density (BMD) and prevalent fractures, or a more severe spondylometaphyseal dysplasia with neonatal fractures, long‐bone deformities, and short stature.^(^ ^2^ ^)^ The cranial sclerotic, circular lesions differentiate this skeletal disorder from other types of primary osteoporosis.^(^ ^2^ ^)^

Sphingomyelin (SM) is an essential structural component in the plasma membrane, and the catalytic role of SMS2 in the phosphate‐yielding sphingolipid metabolism suggests a key role in matrix mineralization.^(^ ^2^, ^3^ ^)^ Our previous basic histomorphometric evaluation of patients' bone biopsy samples depicted an overall decrease in bone volume with a disorganized collagenous network, overall reduced mineral content, and increased heterogeneity in matrix mineralization.^(^ ^2^ ^)^ Although the findings suggested increased osteoclast numbers, further functional analyses found no differences in osteoclast morphology or their resorptive capacity.^(^ ^2^ ^)^ Moreover, *Sgms2* was highly expressed in osteoblasts and osteoclasts in murine cortical bone. In light of these results and the patients' clinical manifestations, we concluded that sphingolipid metabolism has an evident role in bone health and its disturbance a damaging effect on bone material properties. Still, the exact consequences and mechanisms of aberrant SMS2 function in bone homeostasis remain incompletely understood.

We therefore set out to further examine the changes in bone material and dynamic properties in *SGMS2* mutation‐positive subjects. Here we provide a detailed analysis of bone tissue characteristics, including osteocyte and their lacuno–canalicular network characteristics, in bone biopsy samples from two affected adult males. The results reveal severe material defects specifically in bone matrix mineralization, osteocyte orientation and the canalicular network.

## Patients and Methods

### Patients

The study includes two unrelated Finnish subjects previously diagnosed with a heterozygous *SGMS2* nonsense mutation p.Arg50*, identified through whole‐exome sequencing and confirmed by Sanger sequencing, as described.^(^ ^2^ ^)^ The patients' clinical characteristics were briefly reported in Pekkinen and colleagues.^(^ ^2^ ^)^ The mutation introduces a premature stop codon in exon 2, resulting in a truncated SMS2 protein lacking the entire membrane‐spanning core domain. Both subjects were re‐recruited to undergo transiliac bone biopsy, after providing informed consent prior to study enrollment. For patient 2, an earlier sample had been taken in childhood, 15 years prior to present study, and its results have been analyzed and reported before.^(^ ^2^ ^)^ All aspects of the current study were approved by the ethics committee of the Helsinki University Hospital (HUS/1088/2016).

### Clinical studies

Patients' prior clinical and treatment data were reviewed and associated with biopsy data. Biochemical parameters were evaluated on the day of biopsy from peripheral blood obtained in the morning between 8:00 am and 10:00 am, after an overnight fast. Measurements were done at HUSLAB laboratories in Helsinki, Finland. We assessed peripheral blood biochemistry for complete blood count, calcium (P‐Ca) and phosphate (P‐Pi), 25‐hydroxyvitamin D (S‐25‐OH‐D; assessed by chemiluminescent immunoassay [CLIA]; Abbott, Deerfield, IL, USA), creatinine (P‐Cr), parathyroid hormone (P‐PTH; CLIA assay on the IDS‐iSYS fully automated immunoassay system; Immunodiagnostic Systems, Ltd., Bolton, UK), and metabolic bone markers including alkaline phosphatase (P‐ALP), bone formation marker N‐terminal propeptide of type I procollagen (P‐P1NP; CLIA, IDS‐iSYS), and bone resorption marker collagen type 1 cross‐linked C‐telopeptide (P‐CTx; CLIA, IDS‐iSYS).

### Bone biopsies

Both subjects underwent transiliac bone biopsy at Helsinki University Hospital. Biopsy samples were obtained of the anterior superior iliac crest with a trephine inner diameter of 7.5 mm (Rochester Bone Biopsy; Medical Innovations International, Rochester, MN, USA) under local anesthesia and iv sedation. Prior to obtaining the samples, both subjects received tetracycline labelling following a routine protocol: two courses of oral tetracycline (500 mg twice a day for 3 consecutive days), with a 12‐day interval, with biopsy obtained 5 days after the second course.

### Sample preparation

Immediately after biopsy the bone samples were immersed in 70% ethanol for fixation. For analysis of osteocyte lacunocanalicular network (OLCN, see below section 2.7), the samples were cut in two pieces; one part of the sample was stained with rhodamine diluted in 70% ethanol prior to the embedding process for the OLCN analysis, the other part of the sample was used for all other analyses. Both samples were dehydrated in a graded series of alcohol. Subsequently, the undecalcified samples were embedded in methylmethacrylate and 3‐μm‐thick sections were obtained with a hard tissue microtome (Leica SM2500; Leica, Nussloch, Germany). These sections were deplasticized with 2‐methoxyethyl‐acetate and stained with modified Goldner's Trichrome for evaluation of static histomorphometric parameters. Digital images of the sections were obtained with a light microscope (Axiophot; Zeiss, Oberkochen, Germany) equipped with a digital camera (AxioCam HRc; Zeiss). Additionally, the bone matrix organization was observed under polarized light. Using custom‐made macros in ImageJ software (NIH, Bethesda, MD, USA; https://imagej.nih.gov/ij), bone histomorphometry analyses were performed on four randomly chosen areas throughout each bone section. Histomorphometric results were compared to age‐ and sex‐matched controls as reported by Rehman and colleagues.^(^ ^4^ ^)^

The surface of the residual sample block was ground by sandpaper and subsequently polished using diamond suspension (3 μm and 1 μm grain size) on a precision polishing device (PM5 Logitech; Logitech, Glasgow, UK). Dynamic histomorphometric parameters were evaluated by measuring the tetracycline labeling on the surface of the block with confocal laser scanning microscopy. Prior to quantitative backscattered electron imaging (qBEI) the samples were carbon coated (Agar SEM Carbon Coater; Agar, Stansted, UK).

### Bone mineralization density distribution

Bone mineralization density distribution (BMDD) measurements were performed by qBEI as described.^(^ ^5^ ^)^ In brief, the entire cross‐sectional bone sample area was imaged with a spatial resolution of 3.6 μm per pixel using a digital scanning electron microscope (DSM 962; Zeiss) equipped with a four‐quadrant semiconductor backscattered electron detector. The DSM was operated with an electron energy of 20 keV and a probe current of 110 ± 0.4 pA at a working distance of 15 mm. Prior to measuring, the device was calibrated with carbon and aluminum standards of high purity. From the obtained 8‐bit images, the frequency distribution of gray levels was converted to a frequency distribution of Ca concentration given in weight %; that is, the BMDD characterized by the evaluation of five parameters: (i) CaMean (weighted mean Ca content), (ii) CaPeak (most frequently measured Ca content), (iii) CaWidth (full width at half maximum of the BMDD—indicative for the heterogeneity of mineralization), (iv) CaLow (percentage of bone area mineralized below 17.68 weight % Ca—5% percentile of the adult trabecular reference BMDD), and (v) CaHigh (percentage of bone area mineralized above 25.30 weight % Ca—95th percentile of the adult trabecular reference BMDD). Measurements were compared between cortical and trabecular bone in the same patient and between trabecular bone and established trabecular reference values of healthy adults.^(^ ^5^, ^6^ ^)^

### Osteocyte lacunae sections

Osteocyte lacunae were analyzed from qBEI images with a pixel resolution of 0.88 μm obtained using a field emission scanning electron microscope (FESEM) (Zeiss Supra 40; Zeiss) working with 20 keV energy, and a 10 mm working distance. The osteocyte lacunae sections (OLS) density, size, and geometric shape (aspect ratio) were determined as described.^(^ ^7^ ^)^ We used a gray level threshold of 55 (corresponding to 5.2 weight % Ca) to define the OLS. A size cutoff of 200 μm^2^ was chosen to discriminate between osteocyte lacunae and pores of larger size; eg, vascularity, Haversian channels, or cracks. Results were compared with children's reference data (samples used in Blouin and colleagues^(^ ^7^ ^)^) because adult references were not available.

### OLCN

Using a confocal laser scanning microscope (Leica TCS SP5; Leica, Wetzlar, Germany) equipped with an oil immersion lens (HCX PL APO 40× Numerical Aperture (NA) 1.25 oil; Leica), the fluorescent signal from rhodamine was measured in depth of the sample.^(^ ^8^, ^9^ ^)^ A HeNe laser with 543 nm wavelength was chosen for rhodamine excitement and the fluorescence signal was measured in a spectral detection window between 553 and 705 nm with the airy 1 pinhole of 67.93 μm. A stack of two‐dimensional images of 1024 × 1024 pixels with 0.38 μm lateral resolution and 0.25 μm depth resolution was recorded and used to visualize and analyze the three‐dimensional (3D) structure of the network.

## Results

### Clinical data

**Fig 1:**
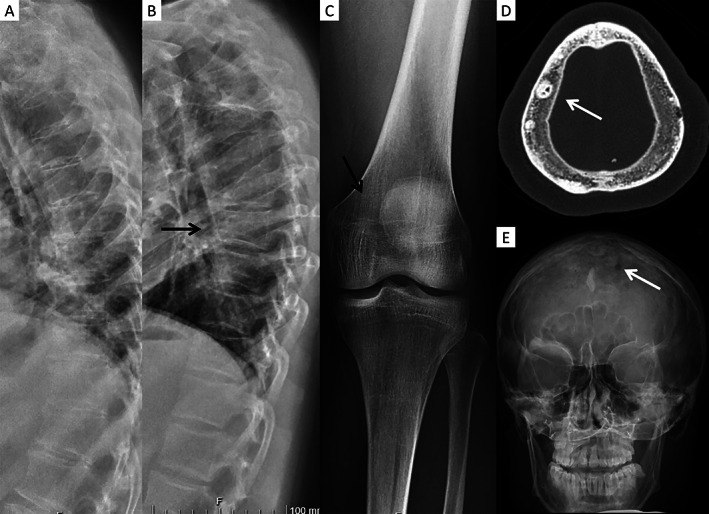
Skeletal radiographs for the two males with *SGMS2* nonsense mutation p.Arg50*. (*A*,*D*) Patient 1, at age 55 years. (*B*,*C*,*E*) Patient 2, at age 27 years. Images show poor and uneven mineralization with visible trabeculation in vertebrae and long bones (black arrows) and multiple sclerotic cranial lesions in skull bones (white arrows).

Patient 1 is a 61‐year‐old male with early‐onset osteoporosis, decreased BMD, and frequent fractures (Fig. 1A and D). He has had several vertebral compression fractures and at least nine long‐bone fractures since early childhood, all resulting from low‐energy traumas. His BMD *T*‐score/*Z*‐score, at the age of 59 years, were −0.8/−1.0 for lumbar spine, −1.0/−0.5 for femoral neck, and −0.6/−1.2 for whole body; no vertebral compression fractures or osteochondrosis were observed in the lumbar spine to account for the normal BMD. He was not treated with osteoporosis medication prior to biopsy. He has received inhaled glucocorticoids for asthma with intermittent, low to moderate dosage, which can be regarded to have minimal impact on bone health.^(^ ^10^ ^)^ Radiographs portray an overall osteoporotic appearance in long‐bones and multiple doughnut‐shaped sclerotic lesions in cranial bones (Fig. 1A and D). Biochemistry on the morning of biopsy showed normal levels for all measured parameters excluding slightly decreased S‐25‐OHD (42 nmol/L) (Table 1).

**Table 1:** Biochemistry in Two Male Patients With *SGMS2* Nonsense Mutation p.Arg50*

Parameter	Definition (unit)	Patient 1: 61‐year‐old male	Patient 2: 29‐year‐old male	Reference range^a^
Hb	Hemoglobin (g/L)	153	153	134–167
P‐Ca	Calcium (mmol/L)	2.28	**2.69**	2.15–2.51
S‐Ca‐ion	Calcium ion (mmol/L)	1.2	**1.31**	1.16–1.3
P‐Pi	Phosphate [mmol/L]	0.89	1.26	0.71–1.53
S‐25‐OHD	25‐hydroxyvitamin D (nmol/L)	*42*	70	>50
P‐Cr	Creatinine (μmol/L)	76	*58*	60–100
P‐PTH	Parathyroid hormone (ng/L)	76	39	18–80
P‐ALP	Alkaline phosphate (U/L)	68	**123**	35–105
P‐P1NP	Intact procollagen I N‐terminal propeptide; bone formation marker (μg/L)	30	59	15–59
P‐CTx	C‐terminal telopeptides Type I collagen (μg/L)	N/A	**0.78**	<0.58

All samples taken in the morning, after an overnight fast, between 8:00 am and 10:00 am. Supranormal values are in bold, subnormal values are in italics.

N/A = not available; P = plasma; S = serum.

^a^ Reference ranges according to HUSLAB laboratories.

Patient 2 is a 29‐year‐old male with childhood‐onset skeletal fragility (Fig. 1B, C and E). He has sustained multiple vertebral compression fractures and altogether 12 long‐bone fractures since early childhood. His BMD *T*‐score/*Z*‐score, at the age of 29 years, were −4.1/−4.1 for lumbar spine and −0.6/−0.4 for femoral neck. He was treated with iv zoledronic acid with dose 0.05 mg/kg twice a year for 2 years between the of ages 15 and 17 years, with the last dose given 12 years prior to biopsy. He had no other long‐term medications. Radiographs show an overall diminished bone mineral content with an uneven mineralization pattern (Fig. 1B, C and E). On previous measurements, his P‐ALP has been elevated, as it was also on the morning of biopsy (123 U/L, reference range 35–105 U/L) (Table 1). Other biochemistry showed slightly elevated calcium (P‐Ca 2.69 mmol/L, S‐Ca‐Ion 1.31 mmol/L) and P‐CTx (0.78 μg/L); other measured parameters were within normal limits.

Both patients have previously been found to harbor the same heterozygous nonsense variant c.148C>T (p.Arg50*) as described.^(^ ^2^ ^)^

### Bone histomorphometry

All histomorphometric results, compared against references,^(^ ^4^ ^)^ are summarized in Table 2. For both patients, structural parameters including bone volume as well as trabecular thickness and number were within normal range (*Z*‐values within ±2). Osteoid thickness (O.Th) was slightly decreased in both subjects (patient 1: −1.80 SD; patient 2: −1.37 SD). Osteoid (OS/BS) and osteoblast (Ob.S/BS) surfaces were markedly increased in patient 1 (+9.03 SD and +2.05 SD, respectively), whereas in patient 2 OS/BS was only slightly increased (+0.98), but Ob.S/BS was significantly reduced (−2.47 SD). Dynamic parameters of bone formation were particularly low in patient 2, specifically mineralizing surface (MS/BS; −2.73 SD), adjusted apposition rate (Aj.Ar; −2.67 SD), and bone formation rate (BFR/BS; −2.05 SD). Consequently, patient 2's mineralization lag time was strongly increased (Mlt: +4.10 SD). Patient 1 showed a moderate decrease in bone formation activity, but a notable increase in mineralization lag time (+8.16 SD). Bone resorption was significantly increased in both subjects as mirrored by extended eroded surfaces (ES/BS; patient 1: +3.45 SD; patient 2: +10.46 SD).

**Table 2:** Bone Histomorphometry Results for Patients 1 and 2 With *SGMS2* Nonsense Mutation p.Arg50*

Parameter, variable (unit of measure), definition	Patient 1 Male 61 years Difference (SD)	Reference (SD) Male 61–70 years	Patient 2 Male 29 years Difference (SD)	Reference (SD) Male 21–30 years
Bone structure				
Spongiosa				
BV/TV (%), bone volume	22.19 (+0.60)	19.2 (5.00)	22.55 (−0.27)	23.90 (5.0)
Tb.Th (μm), trabecular thickness	134.71 (−0.12)	138 (28)	91.83 (−1.82)	141.00 (27)
Tb.N (1/mm), trabecular number	1.65 (+0.38)	1.5 (0.4)	2.46 (+1.90)	1.70 (0.4)
Corticalis				
Ct.Wi (mm), cortical width	**0.78 (−3.82)**	1.2 (0.11)	0.41 (−1.99)	1.28 (0.43)
Ct.Po (%), cortical porosity	4.46		38.60	
Bone formation				
Static				
OV/BV (%), osteoid volume	2.73 (+0.30)	2.4 (1.1)	1.51 (−1.10)	3.60 (1.90)
O.Th (μm), osteoid thickness	4.1 (−1.80)	8.6 (2.5)	4.22 (−1.37)	8.60 (3.20)
OS/BS (%), osteoid surface	**50.33 (+9.03)**	12.4 (4.2)	21.31 (+0.98)	16.10 (5.30)
Ob.S/BS (%), osteoblast surface	**7.45 (+2.5)**	4.7 (1.1)	**0.46 (−2.47)**	5.40 (2.00)
Dynamic				
MS/BS (%), mineralizing surface	3.8 (−1.03)	7.6(3.7)	**2.62 (−2.73)**	7.80 (1.90)
MAR (μm/day), mineral apposition rate	0.7 (+0.55)	0.59(0.2)	0.70 (+0.50)	0.64 (0.12)
Aj.Ar (μm/day), adjusted apposition rate	0.053 (−1.08)	0.28(0.21)	**0.09 (−2.67)**	0.41 (0.12)
BFR/BS (μm/year), bone formation rate at bone surface	9.73 (−0.69)	16.4(9.7)	**6.73 (−2.05)**	18.22 (5.60)
BFR/BV (% /year), bone formation rate of bone volume	14.91 (−0.32)	20.2(16.5)	14.66 (−1.21)	25.40 (8.90)
Mlt (days), mineralization lag time	**77.38 (+8.16)**	17.8(7.3)	**48.78 (+4.10)**	17.60 (7.60)
Bone resorption				
ES/BS (%), eroded surface	**8.67 (+3.45)**	3.5 (1.5)	**16.25 (+10.46)**	3.7 (1.2)
Oc.S/BS (%), osteoclast surface	0.28 (−1.4)	0.7 (0.3)	**1.9 (+4.33)**	0.6 (0.3)
N.Oc/BS (1/mm), osteoclast number	0.1		0.48	

Histomorphometric parameters from patients 1 and 2. Parameters that largely differ from the reference values (difference more than 2 SD) are in bold. Reference values are obtained from Sutter and Stein.^(^ ^10^ ^)^

**Fig 2:**
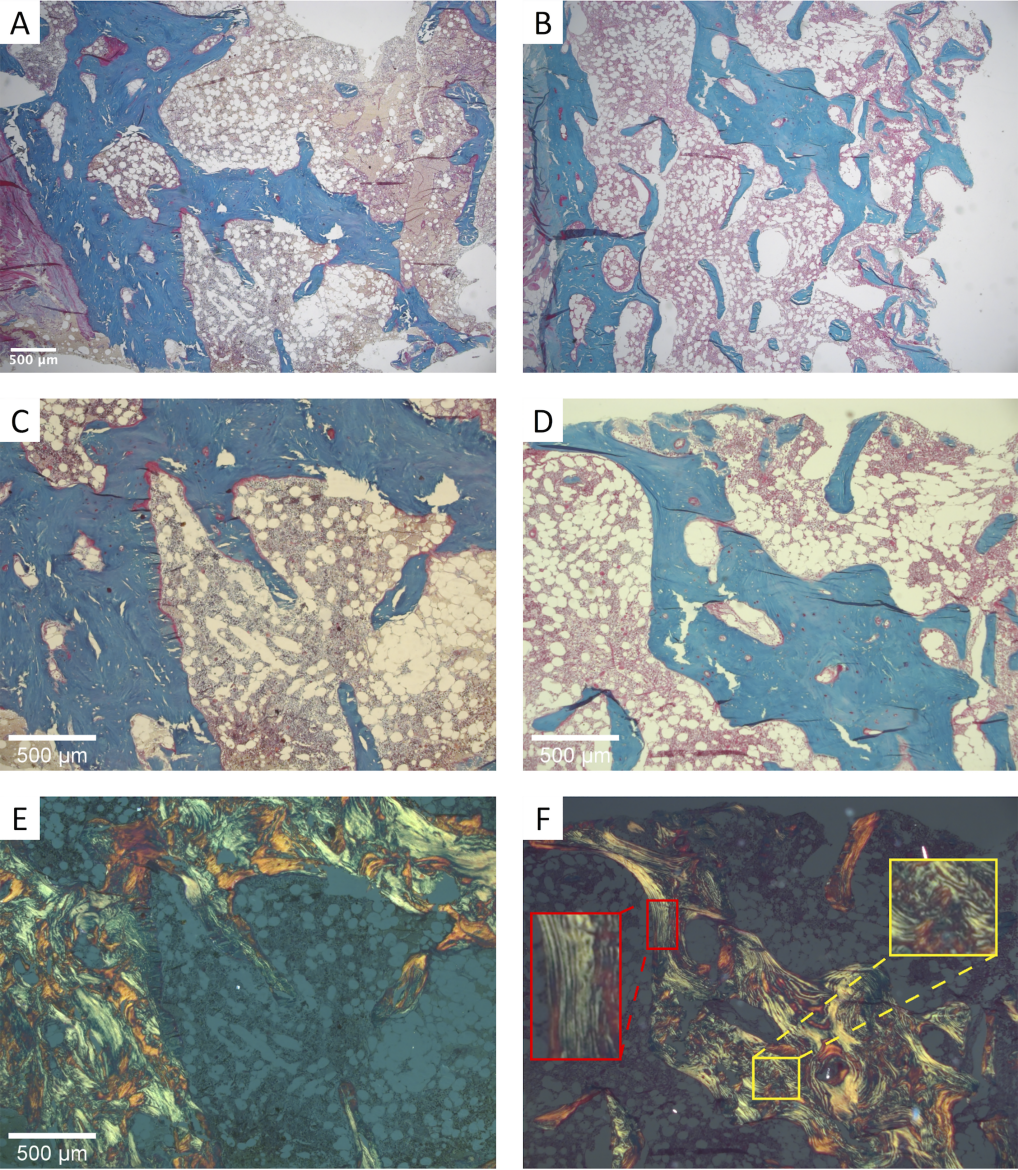
Optical images from bone sections stained with Goldner trichrome from two male patients with a *SGMS2* nonsense mutation p.Arg50*. Overview images from sections of (*A*) patient 1 and (*B*) patient 2. Close‐up images of (*C*) patient 1 and (*D*) patient 2 show the presence of osteoid (red staining) apposed on the mineralized bone (green staining). (*E*,*F*) The corresponding images taken with polarized light and showing large amount of disorganized/woven bone. The insets in *F* show regions of lamellar (red) and woven bone (yellow).

**Fig 3:**
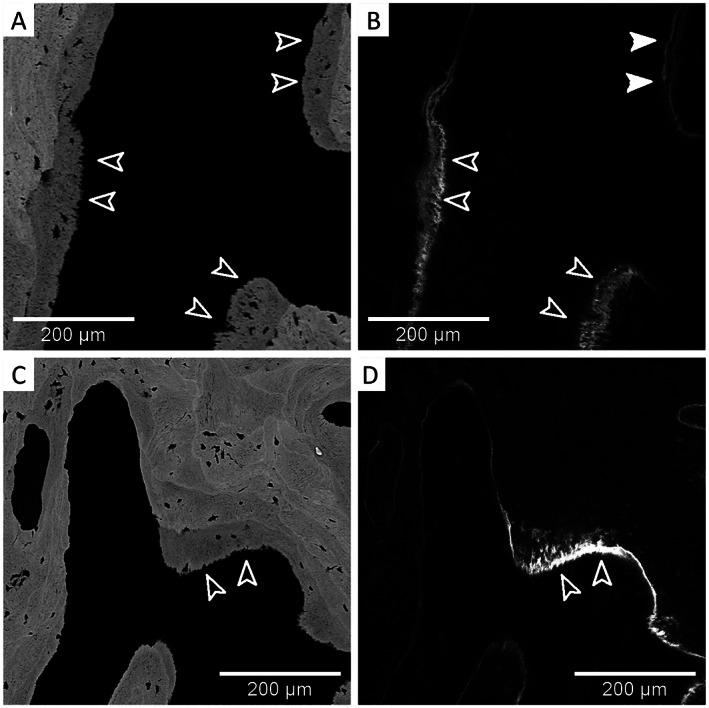
qBEI images from bone biopsy samples from two male patients with *SGMS2* nonsense mutation p.Arg50*. (*A*) Patient 1 and (*C*) patient 2, and the corresponding images from confocal laser scanning microscope showing the tetracycline labelling in (*B*) patient 1 and (*D*) patient 2. Open arrows in *B* and *D* show blurred labelling, whereas full arrows indicate a double label. Arrows in *A* and *C* show frazzled mineralizing bone surfaces.

Polarized light microscopy images of Goldner‐stained sections, particularly from the cortex, revealed the presence of non‐lamellar bone with a disordered and chaotic arrangement of collagenous fibrils (Fig. 2A‐F). Last, in line with the histomorphometric parameters, tetracycline labels, indicating the movement of the mineralization front, were often blurred and partly inadequately separated; thus, the mineralizing bone surface appeared frazzled in both patients (Fig. 3A‐D).

### BMDD

#### Cortical bone is more mineralized than trabecular bone

**Fig 4:**
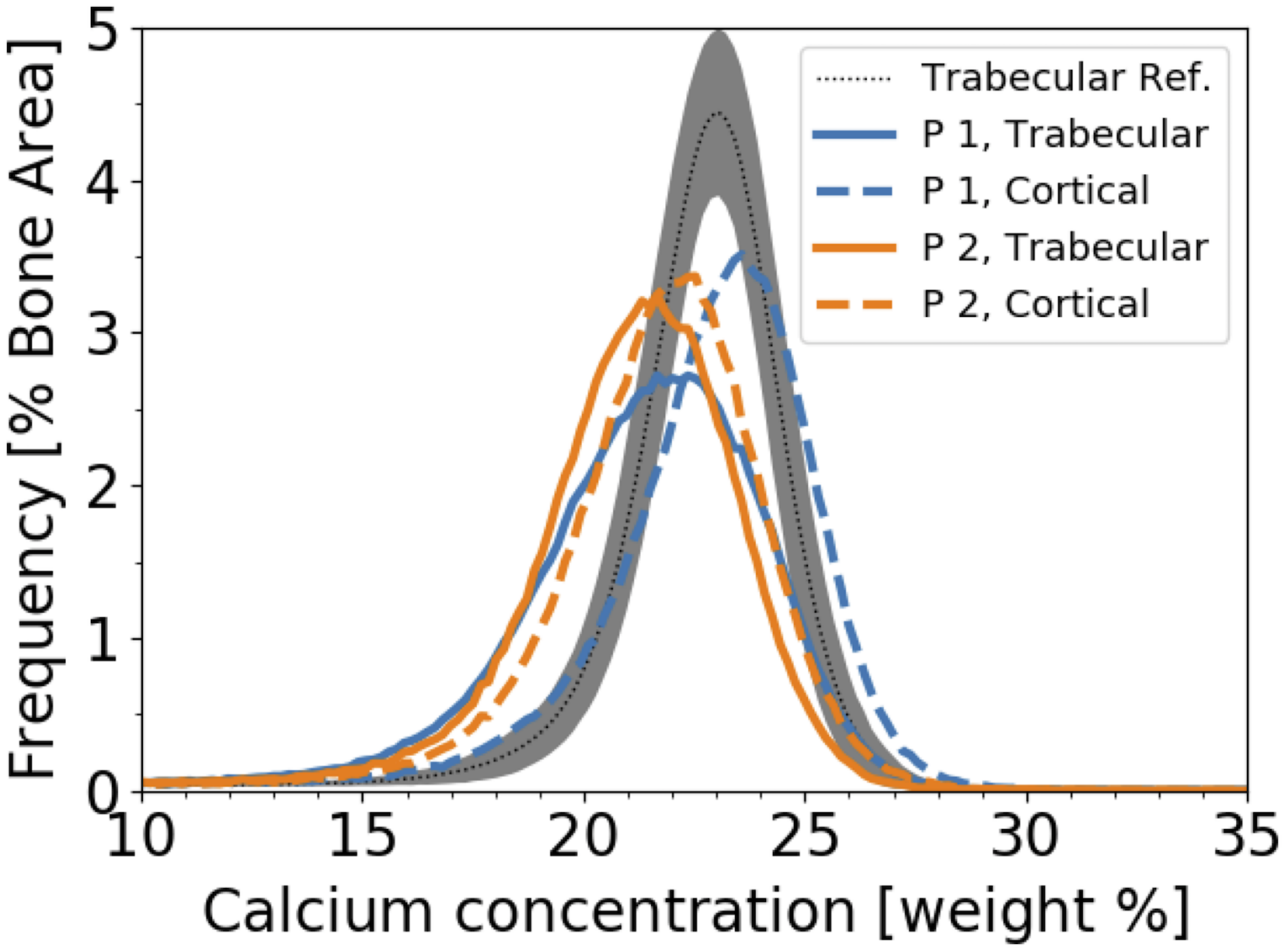
BMDD from cortical and trabecular bone in two male patients with *SGMS2* nonsense mutation p.Arg50*. Patient 1 (blue lines) and patient 2 (orange lines) compared to a BMDD reference (dotted line is the mean, gray area is the ±1 SD area) from 52 healthy controls.^(^ ^5^ ^)^ BMDD = bone mineralization density distribution.

In both patients, mineralization density was higher in cortical than in trabecular bone (Fig. 4, Table 3). This is reflected in increased values of CaMean (patient 1: +7.3%; patient 2: +3.1%), CaPeak (patient 1: +6.4%; patient 2: +2.4%), and CaHigh (patient 1; +169.2%; patient 2: +101.8%) in the cortical compared to the trabecular compartment. Moreover, CaLow (patient 1: −46.2%; patient 2: −22.5%) and the heterogeneity in mineralization were decreased (patient 1: −26.5%; patient 2: −7%).

**Table 3:** Bone Mineral Density Distribution Outcomes From Cortical and Trabecular Bone of Two Male Patients With an *SGMS2* Nonsense Mutation p.Arg50*

	Cortical bone		Trabecular bone Difference from reference (SD)		
Parameter	Patient 1 Male 61 years	Patient 2 Male 29 years	Patient 1	Patient 2	Trabecular reference
CaMean (wt%Ca)	22.39	21.27	*20.86 (−2.98)*	*20.63 (−3.49)*	22.2 ± 0.45
CaPeak (wt%Ca)	23.40	22.01	*22.0 (−2.41)*	*21.49 (−3.72)*	22.94 ± 0.39
CaWidth (Δ wt%Ca)	4.33	4.51	**5.89 (+7.47)**	**4.85 (+4.41)**	3.35 ± 0.34
CaLow (% Bone area)	6.42	8.17	**11.93 (+4.46)**	**10.54 (+3.57)**	4.93 ± 1.57
CaHigh (% Bone area)	12.68	4.48	4.71 (−0.25)	2.22 (−1.00)	5.55 ± 3.32

Bone mineral density distribution values were obtained from qBEI measurements. Parameters that significantly differ from the reference values (>2 SD) are indicated: supranormal values are in bold; subnormal values are in italics. Reference values are obtained from Roschger and colleagues.^(^ ^5^ ^)^

Ca = calcium; wt = weighted.

#### Trabecular bone is less mineralized than healthy references

In both patients, mineralization density in trabecular bone was lower than in controls (CaMean; patient 1: −2.98 SD, patient 2: −3.49 SD; CaPeak; patient 1: −2.41 SD; patient 2: −3.72 SD) (Fig. 4, Table 3). Consistently, CaLow was markedly increased (CaLow; patient 1: +4.46 SD; patient 2: +3.57 SD), whereas CaHigh was slightly reduced (CaHigh; patient 1: −0.25 SD, patient 2: −1.00 SD) compared to the reference. The heterogeneity in mineralization CaWidth, was increased (CaWidth; patient 1: +7.47 SD; patient 2: +4.41 SD).

### OLS and the OLCN

**Fig 5:**
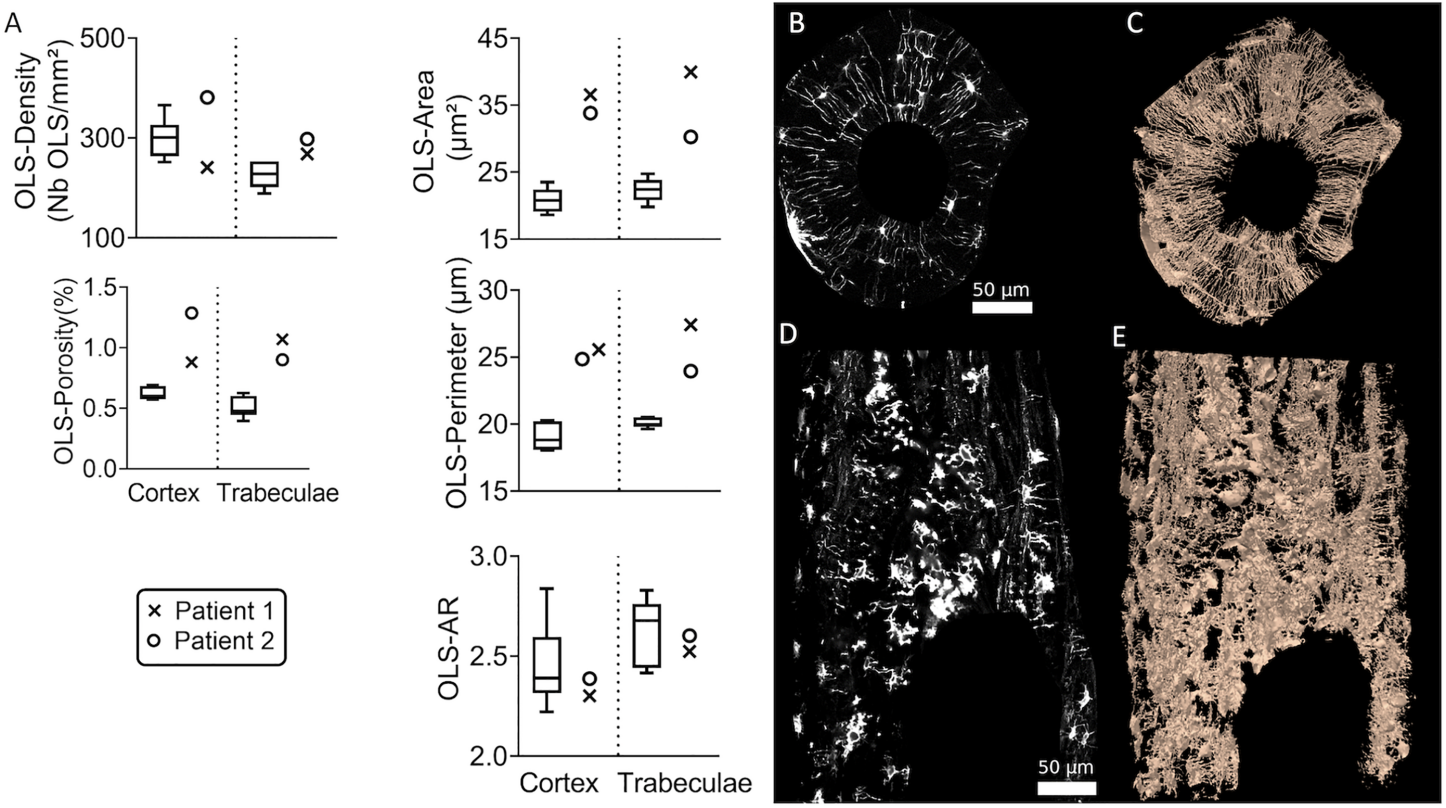
Osteocyte characteristics in two males with *SGMS2* nonsense mutation p.Arg50*. (*A*) Osteocyte lacunae sections data obtained from qBEI images. The porosity due to the osteocyte lacunae is extremely increased in both patients mainly due to a tremendous increase of the lacunae size. (*B*) An optical section obtained with a confocal laser scanning microscopy from a cortical bone sample obtained in a healthy 57‐year‐old female and (*C*) the 3D reconstruction from the image stack containing this optical section. The OLCN is well‐organized around an osteon with small lacunae placed circumferentially and canaliculi oriented to the center of the osteon. In contrast, the optical section (*D*) and the 3D reconstruction (*E*) obtained in patient 2 show large lacunae and a disorganized network.

The measurement of the OLS from qBEI images showed high porosity in both biopsy samples, which was mainly due to a remarkably large OLS size (area and perimeter) compared to previously reported data obtained from six healthy children.^(^ ^7^ ^)^ In addition, in both patients the OLS aspect ratio was found in the low range (particularly in the cortex), indicating a more circular shape of the OLS in the patients as compared with healthy children (Fig. 5*A*).

Figure 5*B*‐*E* visualize the OLCN as obtained from rhodamine stained samples with confocal laser scanning microscopy. For comparison, one representative slice and a reconstructed 3D representation of the network from patient 2 was contrasted with the results from a healthy individual. Compared to the organized, well‐aligned canaliculi that typically run radially in a plane perpendicular to the axis of the osteon in normal cortical bone (Fig. 5*B*,*C*), both samples exhibited a haphazard, short‐ranged network that lacked any continuity, orderliness, and symmetry.

Taken together, the *SGMS2* mutation exerts a damaging effect on the synthesis and organization of bone extracellular matrix with, or consequently, a severely disturbed OLS shape and size and OLCN network.

## Discussion

Here we have expanded the characterization of the tissue‐level bone pathology in patients with primary osteoporosis due to a pathogenic variant in the *SGMS2* gene. The study assessed transiliac bone biopsy samples from two unrelated male patients with the same *SGMS2* nonsense variant p.Arg50* and consequent childhood‐onset skeletal fragility. The results show low and heterogeneous bone matrix mineralization that extends to both cortical and trabecular bone. In addition, matrix collagenous fibrils are chaotically organized. Most significantly, the osteocyte lacunae and their canalicular network were severely disturbed. These findings provide further insight into the bone tissue changes resulting from abnormal function of SMS2.

Bone histomorphometry revealed, in both patients, a striking increase in mineralization lag time (Mlt) in association with extended osteoid surface and low mineralizing surface. This finding is consistent with the report by Robinson and colleagues^(^ ^11^ ^)^ and indicates a mineralization defect in affected patients. In consequence, qBEI measurements showed a shift in the BMDD curve toward reduced bone matrix mineralization and an increase in width indicating increased heterogeneity of mineralization. Interestingly, and contrary to normal bone tissue,^(^ ^12^ ^)^ the cortical compartment showed higher mineralization than the trabecular compartment in both patients. This can be explained by the larger amount of woven bone observed in the cortex, because woven bone has a higher mineral content than lamellar bone.^(^ ^13^, ^14^ ^)^ Of note, BMD measurements were inconsistent with in‐depth bone tissue analyses, further underlying the limitations of DXA in assessing bone quality in mineralization defects.^(^ ^15^ ^)^

These results highlight the crucial role of sphingomyelin metabolism in bone matrix organization and mineralization. Indeed, SMS2 uses phosphatidylcholine and ceramide to generate sphingomyelin (SM), which in turn serves as a substrate for sphingomyelin phosphodiesterase 3 (SMPD3) to yield phosphocholine and, further, phosphate for mineralization. Correspondingly, the key role of this pathway in matrix mineralization has been demonstrated in in vivo studies. Both the *fro/fro* mice, with a chemically induced deletion in the *Smpd3* locus, and mice deficient in PHOSPHO1, an enzyme also downstream of SMS2, display severe skeletal dysplasia depicted as abnormal cartilage development and poor matrix mineralization.^(^ ^16^, ^17^, ^18^ ^)^ For Smpd3, Coleman and colleagues^(^ ^19^ ^)^ further demonstrated that the mineralization defect in the *fro/fro* mouse is not solely due to a defect in cell‐regulated osteoid mineralization but is also a consequence of deposition of abnormal matrix that is insufficient for crystal maturation, although these results would insinuate a similar link for SMS2 in matrix organization and mineralization. We have previously observed comparable levels of endogenous SMS2 in fibroblasts derived from *SGMS2* mutation‐positive patients compared with control samples^(^ ^2^ ^)^ and hypothesized that the underlying cause is not a reduction in total amount of SM but rather a disturbance in SM metabolism at the plasma membrane. These could indicate a different course of action for SMS2 in matrix maturation and together suggest that abnormal function of the mutated SMS2 alters collagen synthesis directly, leading to irregular lamellation and organization of collagenous fibrils and matrix mineralization.

It is noteworthy that patient 2 had an earlier biopsy taken at the age of 15 years, 14 years prior to the biopsy analyzed in the present study,^(^ ^2^ ^)^ allowing further insights into bone tissue changes introduced with aging. Comparison of earlier findings with the present biopsy showed that whereas the bone volume slightly increased (BV/TV +18%), osteomalacia improved considerably (OV/BV −80%, O.Th. −60%, OS/BS −44%) to nearly normal levels, although osteoid surface remained elevated. Concomitantly, we observed an increase in CaMean in the BMDD (+7.6%). The shape of the BMDD (characterized by its width) remained almost unchanged. This increase in mineralization corresponds to the expected effect of aging (from adolescence to adulthood), whereas the unchanged width of the curve suggests a persistent matrix and/or mineralization abnormality.

Polarized light microscopy showed that lamellar orientation was highly disordered. In both samples we observed the presence of a large portion of woven bone and, consequently, fluorescent double labels could not be separated because the disordered collagen fibrils caused pronounced smearing of the labels. This is in contrast to properly mineralizing lamellar bone exhibiting sharp double labels and smooth bone surfaces. The finding of a large portion of woven bone (especially in the cortex) is consistent with our earlier findings,^(^ ^2^ ^)^ but contradictory to results in another study reporting the presence of well‐aligned lamellae in the cortex.^(^ ^11^ ^)^ Nevertheless, regions with organized lamellae can also be found in our patients (Fig. 3).

In addition to the disordered collagenous apposition, osteocyte lacunae appeared abnormal in size (too large) and the lacunocanalicular network extremely distorted and short‐spanned. This is in contrast to lamellar bone, in which canaliculi typically run radially in a plane perpendicular to the axis of the osteon, emerging from osteocyte lacunae and forming a dense network that ensures sensitivity and tight communication between neighboring osteocytes, other bone cells, and vasculature. This disturbance in OLCN might reflect the large amount of woven bone. A disordered arrangement of collagen does not allow for a regular network to evolve.^(^ ^20^ ^)^ The large amount of woven bone may also explain the abnormal size and shape of osteocyte lacunae; the normal arrangement of the long axes off osteocyte lacunae in parallel with collagen orientation could be compromised in the highly disturbed surroundings.

Whether the osteocyte pathology is secondary to the distorted surrounding matrix or also a direct outcome of aberrant SMS2 function in osteocytes is unclear. Few reports have suggested a functional role for SM metabolism in osteocytes. For example, Zhang and colleagues^(^ ^21^ ^)^ showed that sphingosine‐1‐phosphate (S1P), a substrate of ceramide deacetylation, modulates osteocyte cellular responses to mechanical loading from oscillatory fluid flow.^(^ ^21^, ^22^ ^)^ This is supported by the notation that lipid rafts—sphingolipid‐enriched plasma membrane microdomains—serve a key role in mechanosensing and mechanotransduction and that disruptions in osteocytes' plasma membranes initiate their mechanosensation.^(^ ^23^, ^24^, ^25^ ^)^ Nevertheless, because no study, so far, has explored the role of SMS2 in osteocytes specifically, and the data on other sphingomyelin pathway components in osteocytes is also scarce, the exact role of sphingomyelin metabolism in osteocytes remains unknown.

We acknowledge that our study has certain limitations, primarily concerning the low number of studied samples and the cross‐sectional nature of the study. A greater number of studied samples, ideally in parallel with samples from patients harboring different *SGMS2* mutations, and at several time points, could have perhaps exposed other marginal findings in greater detail. The use of osteocyte lacunae sections from healthy children as reference for comparison is certainly not optimal but was necessary because no appropriate adult data is available. Our previous measurements in two healthy women were very similar to the children's values,^(^ ^26^ ^)^ further justifying the use of the mentioned reference data. Also, the comparison of the OLCN of patient 2 (male, 29 years) with the OLCN of a healthy female aged 57 years is not ideal. Although the two samples are neither gender‐ nor age‐matched, they still give a qualitative impression on the disturbed nature of the OLCN in patient 2. Clearly, for quantitative comparisons of, eg, network density, age‐ and gender‐matched references would be highly desirable. Also, although neither patient exhibited systemic disturbance in phosphate metabolism, quantification of tissue level concentrations of phosphate and other components of the SM pathway would be useful and warrants further studying. Although some disparities in results were observed between the two patients, possibly accounted for by the age difference between the patients, an evident trend was observed. We also acknowledge the rarity of *SGMS2* mutation‐positive subjects and the invasive nature of obtaining a bone biopsy sample and therefore consider our data unique and valuable.

In conclusion, we report a severe negative effect of the *SGMS2* mutation on bone material quality including a strongly disturbed bone matrix mineralization and a disorganized collagen fibril arrangement. This is due to an impaired bone cell activity. Alongside, or perhaps in consequence, the shape, orientation, and organization of osteocytes and their interconnected network are deformed. The role and significance of the mutated SMS2 specifically in osteocytes require further studies. These results will hopefully serve in the collective effort to better our understanding of the central genetic and molecular players in skeletal health and disease, specifically in sphingolipid‐related pathways.

## Conflict of Interest

The authors declare no conflicts of interest.

### PEER REVIEW

The peer review history for this article is available at https://publons.com/publon/10.1002/jbm4.10537.
